# When is work a cause of early retirement and are there any effective organizational measures to combat this? A population-based study of perceived work environment and work-related disorders among employees in Sweden

**DOI:** 10.1186/s12889-020-08865-5

**Published:** 2020-05-19

**Authors:** Kerstin Nilsson

**Affiliations:** 1 Division of Occupational and Environmental Medicine, Lund University, Medicon Village, Scheelevägen 8, By 402A, SE-223 81 Lund, Sweden; 2 Division of Public Health, Kristianstad University, SE-291 88 Kristianstad, Sweden

**Keywords:** Public health, demography, retirement, Occupational health, Work environment, Age management, Older worker, Age management, swAge, Sustainable working life

## Abstract

**Background:**

The ageing workforce has an impact on public health. The aim of this study was to evaluate work-related disorders, work tasks and measures associated with the possibility of working beyond 65 years of age or not.

**Method:**

The data comprised two sample surveys based on the Swedish population: the Survey of National Work-Related Health Disorders, and the National Work Environment Survey.

**Results:**

A logistic regression analysis showed that an active systematic work environmental management in the workplace was a statistically significant association with whether individuals could work in their current occupation until 65 years of age (OR 1.7). The final multivariate model stated that whether individuals could work until 65 years was associated with bodily exhaustion after work, frequent feeling of the own work effort being insufficient at the end of the day, experience of the work as restricted and with a lack of freedom, working alone and at risk of unsafe or threatening situations, and generally feeling dissatisfied with the work tasks. Women-dominated workplaces were more highly associated with both male and female employees not being able to work until age 65 (OR 1.6).

**Conclusion:**

Deficiencies in the working environment seems to be a threat to the public health. An active systematic work environmental management in the workplace increases the possibility to extend the working life. Tools for managers, like the swAge-model, to easily perform active systematic work environmental controls could therefore be a possible way to decrease the risk of work injury as well as increase the possibility for a sustainable extended working life.

## Background

The demographic situation and development with an increased number of elderly citizens is an important factor for the public health and in a nation’s economy. The biggest economic hurdle to economic advance in enterprises, organizations and societies right now is that the workforce is in a critical situation due to the demographic changes in most countries. Fewer people in the workforce need to finance more people in retirement. The old age dependency ratio is increasing in most countries [1]. Giving each worker a second computer does not double his output in a working day. More working hours are needed in the economy to handle the economic burden of an increased number of elderly people in the population. As a result of all this, governments need to make it possible for more people to work until an older age. Therefore, making working life sustainable for people until older ages is a key issue in societies, governments, enterprises and organizations eager to force economic advance. In this, it is important to look not only at an individual’s chronological age in postponing the retirement age.

### Ageing societies and the health situation

The average life expectancy is increasing, which is a positive sign. The average life expectancy is 80 years of age in 34 of the world’s countries today, compared to 19 countries in 2005 [1]. However, since the number of healthy years of living generally have not increased in the EU between 2010 and 2016, this means that the health of the ageing population also poses a societal challenge when work life participation needs to be extended until an older age as a result of the changing demography. In Sweden, it is estimated that 30% of the population will be 60+ years of age in 2050, compared to 20% in 2000. Even higher numbers are presented in other European Union (EU) countries. To maintain the welfare system in the ageing society, the number of working hours in the economy needs to be increased, and through this, citizens need to be motivated to work until an older age [1]. The increasing number of elderly citizens also presents an increased risk of care needs and puts strain on health and hospital care. It is also described as a social and economic tragedy common to virtually all the Organisation for Economic Co-operation and Development (OECD) countries that too many workers leave working life and the labor market permanently due to health problems or disability, and few people with reduced work capacity manage to remain in employment [1]. This is also a paradox because the most physically demanding work tasks and work activities nowadays are performed by technical solutions. However, sick leave due to mental disorders is increasing in the societies. The most common reasons for sick absence in 2018 in Sweden were anxiety and depression (46%, mostly common among women 30–44 years) and musculoskeletal pain and diseases (18%, mostly common among men 50–65+ years) [2]. However, society also needs to consider and reflect on the possible effects of an extended working life. The gaps between different groups’ life expectancy and work life participation have increased over the last year, partly caused by differences in working environment. Due to these increased gaps, a postponed retirement age could pose a possible threat to healthy ageing due to the risks in the work environment and the work tasks in some professions. We need to develop new knowledge concerning the work environment and work tasks that are associated to longer working life and change this trend.

### Theories on how to promote a sustainable working life for all ages

In action to hinder and prevent ill-health and economic suffering among individuals, organizations and societies it is of importance to nudge individuals’ choices towards convenient solutions and decisions that increase health, wealth and happiness [3]. That is also a matter in the work situation in order to increase the possibility for more elderly to work in an extended working life. Managers and organizations also need to be nudged and have access to easily managed measures in order to increase their employees’ health, wellbeing at work and make the work environment more sustainable for all ages. The swAge model (sustainable working life for all ages) is an easily managed tool for this that describes predictors for work life participation in relation to age and determinants of a sustainable working life. The swAge model is used in systematic work environment management performed in organizations to promote the possibility of extending a sustainable working life. The swAge model defines the coherence between different age definitions and nine determinant themes of work life participation, which are sorted into four spheres of measurement [4–6] and described below. To progress toward a sustainable working life in which more people both would be able to and want to work until an older age, it is important to understand ageing in relation to work. The chronological age definition is important when regulating social benefit systems [7]. However, factors associated to biological, social and cognitive ageing are of greater importance when it comes to being able to participate in an extended working life [4, 5]. In the swAge-model, areas of significance to our biological ageing and its effect on individuals being able to and wanting to be part of the workforce until a higher age are: self-rated health, diagnoses, chronic diseases and injuries [8]; physical workplace environment with heavy workload, monotonous and repetitive motions, climate, air, chemical health hazards and accidents [9–11]; mental workplace environment and stress, demands, control, threats and violence [12]; working hours, work pace and possibility of recuperation [14]. The social age definition and its’ impact on being able to and wanting to participate in an extended working life is related to the individual’s partner/family, spare time, and social environment [15–17]; the organizational climate in the workplace relating to leadership, attitudes, discrimination, social support and participation; and how elderly are perceived as co-workers [18]. The cognitive age definition and its’ impact on stimulating, fulfilling and meaningful tasks and activities at work [12, 19], as well as knowledge, skills and competence [20]. Leaving working life is about four considerations related to the four measure spheres in the swAge-model: i) personal health in relation to one’s physical work environment, mental work environment and the possibility of recuperation from the working hours and work pace, which are associated to biological ageing; ii) one’s personal finances in relation to the possibility of supporting themselves and pay for food and shelter through work, the social insurance system and pension system, which is associated with chronological ageing; iii) the circumstances for inclusion, i.e. social participation and social support, or discrimination and disregard at work from management, colleagues, etc. in relation to the possibility of social inclusion with family, friends and leisure activities, which is associated to social ageing; iv) one’s work motivation, stimulation, possibility of development and the ability to use their knowledge in the tasks and activities at work or in leisure time, which is associated to cognitive ageing. The swAge model also describes the differences between being able to and wanting to work until an older age [4, 5, 17]. The conditions required to both be able to and want to work are; sufficient health, financial incentives as well as circumstances outside work with a family situation and/or partner that are conducive to work. Furthermore, being able to work requires a good physical working environment; a good mental working environment; appropriate working hours, work pace and time for recuperation; and proper knowledge and competence. Whether an individual wants to work until a higher age depends on the manager, the leadership, the participation and social support at work, as well as the core of the work with meaningful, stimulating and self-fulfilling tasks and activities [4–6, 18].

Although the chronological age definition is solely related to the social benefit systems’ influence on the individual’s financial situation and when they can afford to retire [4, 5, 13], rather than the individual’s working ability and health, chronological age is still the most cited age definition in the discussion of an extended working life. It is in the interest of governments, societies, organizations and enterprises, as well as individuals, to make working life sustainable and healthy. The effect of the work situation, the work environment and the work tasks on an individuals’ biological ageing, and vice versa, is therefore of great interest in the intention of finding out and developing sustainable measures on the organizational and enterprise level as well as on the governmental level for a sustainable working life for all ages.

### Aim

The objective of this study was to go beyond the chronological age and focus on work-related factors related to whether individuals can work to an older age or whether they leave working life early in population-based surveys with Swedish citizens. The aim was specifically to investigate the possibility to work beyond 65 years of age among men and women associated to their work environments, their work tasks, work-related disorders. The aim was also to investigate whether systematic work environmental management in the workplace as well as access to occupational health care services increase the possibility of working beyond 65 years of age.

## Methods

### Study population and questionnaires

The study population in this study comes from two surveys based on the Swedish population: The Survey of National Work-Related Health Disorders [21], and The National Work Environmental Survey [22]. The random sample data was collected and handled by Statistics Sweden from the Swedish National Labor Statistics and the Swedish Population Register. The inclusion criterion was all employees in Sweden who had an occupational definition, i.e. the population were registered citizens in Sweden and gainfully employed (including employed in their own incorporation and enterprise). The questionnaires were developed and conducted by Statistics Sweden and followed up on the work environment, inconvenience of work accidents and inconvenience of other conditions. Based on these causes of inconvenience, more detail is required on what caused the issues. The variables in the survey also describe different types of actions. Together they form the issues the surveys’ main variables of interest, which intends to be used to describe health problems caused by work in Sweden. The survey and data were conducted and collected 2011–2012 and the data were collected via structured questionnaire-based telephone interviews of the study population. The response rate was 90%. The data from these surveys was also internationally reported and delivered to Eurostat according to the EU regulation, and delivered to OECD to be a part of the OECD Labour Force Statistics.

### The survey of National Work-Related Health Disorders

The Survey of National Work-Related Health Disorders measures the stock of both new and older work-related problems over the course of a year in the Swedish workforce population (15–74 years of age). Respondents aged 31–64 years, and representing all professions in Sweden, were randomly selected by Statistics Sweden to participate in this study. This gives a study population of 13,174 respondents (6297 men and 6877 women) with a median age of 47 years.

### The National Work Environment survey

The National Work Environment survey is based on the Swedish workforce population and describes working conditions and the working environment in Swedish working life. For analysis regarding factors associated to retirement, only participants aged 50–64 years were randomly selected by Statistics Sweden. This because it was only that age group who had answered the question “Do you suppose that you can work until 65 years in your current occupation?” Therefore, this part of the analysis in the paper included 3839 respondents (1780 men and 2059 women).

### Statistical analysis

The investigation method used in the analysis of the Survey of National Work-Related Health Disorders comprised frequency tables and cross tabulation. The outcome regarding different work-related health disorders and work-related demands is shown in percent, as well as in percent of men and women respectively in the results section below. Statistical significance was investigated by Pearson’s chi-squared test (if *p*-value < 0.05).

The statistical method logistic regression analysis was used to investigate if the respondents, who represent the Swedish population, think they could work until 65 years of age in their current occupation in association to whether there was a systematic work environment management at the workplace, whether the workplace had occupational health care, and to different work environmental and work task challenges.

The swAge model [4–6] was used to set and model the analysis of the National Work Environment Survey in this study. Before the logistic regression analysis and modelling, the 23 survey questions/items with relevance for the study objectives were sorted into six areas with importance for work life participation and described in the swAge model. The areas were: (i) physical work environment (7 statements), (ii) mental work environment (5 statements), (iii) work pace and working time (3 statements), (iv) management and social support in work (3 statements), (vi) motivation and work satisfaction (3 statements), and (vii) competence and possibility of skill development (2 statements). These areas were six of the nine areas that were stated as important for work life participation and a sustainable working life for all ages in earlier research and in the theoretical swAge model. The three areas included in the swAge model but not included in this analysis are: personal health, personal finances, and family and social situations. However, the six included areas are important parts of the work environment and measure areas for a sustainable working life, and therefore the possibility of analyzing a represented portion of Swedish employees was determined to be very good.

Originally the response options in the questionnaire were different for different questions, e.g. in some questions the options were “yes” or “no” but the questions regarding the work environment were “fully agree”, “agree to some extent”, “disagree to some extent” and “fully disagree.” For the analysis, the answers to each question/item were sorted and the work environment variables were dichotomized into two categories: agree/yes and disagree/no.

Logistic regression models generating odds ratios (OR), 95% confidence intervals (95% CI) and *p*-values were used to investigate the items statistically associated with the outcomes “I can work until 65 years in my current occupation.” For the outcomes, we used the following analytical strategy:

1. Analyses within each of the six areas: The analysis started with univariate analyses, i.e. evaluation of the associations for one item at a time. In the second step, the item with the lowest *p*-value (if *p* < 0.05) was kept. In the third step, the remaining items were subsequently added, one at a time, to test the robustness of the model. This procedure continued for as long as the *p*-values for all included items were *p* < 0.05.
2. Analyses including all six areas. The analysis started by including the selected items from areas (i) and (ii) in a multivariate model (i.e., the areas “physical work environment” and “mental work environment”). The items with *p*-values < 0.05 were kept in the model to the next step, in which we also included the selected items from area (iii). This procedure continued until all six areas were included in a final model. After that, the discarded items from the six areas were tested, one at a time, in the final model, to check once more if the model was robust.

### Ethical consideration

The research ethics were tested and approved by Statistics Sweden (SCB). Our research group applied and get approval to perform this study and the analyses of the population surveys by the Swedish Ethical Review Authority (Dnr. 2013/11). After injury testing according to the Public Procurement and Secrecy Act, SCB issued access for our research group to microdata for research and statistical purposes. We received data delivered via MONA (Microdata Online Access), Statistics Sweden’s standard tool for making microdata available, and processing the analysis via the internet without microdata leaving SCB.

## Results

### Statements in the work environment survey associated with if people 55–64 years of age could work until 65 years in their current occupation

The analysis of the Swedish National Work Environment Survey found that a total of 91.3% of participants thought they could work until 65 years of age in their current occupation (chi^2^*p* < 0.001, 93.5% of the men and 89.4% of the women). A cross-tabulation analysis was performed of individuals identified as working in female- or male-dominated workplaces and of the expectation to work until the ordinary retirement age of 65 years. The analysis found that 67.4% of those who stated that they could not work until ordinary retirement age worked in female-dominated workplaces, compared to 32.6% in male-dominated workplaces (chi^2^*p* < 0.005). A logistic regression analysis showed that at workplaces with higher numbers of women than men, i.e. women-dominated workplaces as opposed to male-dominated workplaces, a larger number of employees reported that they not could work until 65 years than vice versa (OR 1.6, 95% CI 1.2–2.2). A subsequent analysis of men’s and women’s answers separately distinguished no statistically significant difference between whether it was a woman or a man who reported the impossibility of working until 65 years of age in female-dominated workplaces.

### The shape and performance of the work tasks in association with an extended working life

The work tasks are important to whether or not people can go on working until an older age. A logistic regression analysis found that whether or not respondents aged 55–64 thought they could go on working in their current occupation was highly associated with the statement “given my age, it is hard to manage the work tasks I have today” (Total: OR 8.5, (95% CI 6.1–11.9); Men: OR 9.5, (95% CI 5.5–16.3); Women: OR 7.8, (95% CI 5.1–12.0)).

Due to the importance of the work tasks to an extended working life, the shape and performance of work tasks were analyzed further in the analysis. To set the analysis, the 23 items from the survey were grouped for the analysis within different themes, based on the themes in the theoretical swAge model (for further description, see the introduction and method chapters).

The logistic regression analysis with simple and multivariate models showed that within the theme Physical work environment, the variables “bodily exhaustion after work” and “strenuous working poses” were mostly associated with whether the respondents could work until 65 years of age or not (Table 1). Bodily exhaustion was mostly associated with women and strenuous working poses was mostly associated with men when the sexes were subsequently split in the multivariate model of the theme.

**Table 1:** Distributions regarding “can work until age 65 in the current occupation” outcome included in the final multivariate model and the corresponding odds ratios (ORs) and 95% confidence intervals (CIs) obtained from logistic regression. Increased ORs for an item indicate individuals’ decreased possibility to work until age 65 in their current occupation. (* = *p* < 0.05; ** = *p* < 0.005)

Theme	Statement	Groups	Univariate estimate		Included in the final multivariate model of the theme	
			OR	95% Cl	OR	95% Cl
Physical work environment	Bodily exhaustion after work	Total	4.7*	3.4–6.6	4.3**	2.7–6.8
		Men	4.2*	2.4–7.3	3.0**	1.4–6.5
		Women	4.8*	3.2–7.3	5.0**	2.8–8.9
	Strenuous working poses	Total	3.3*	2.3–4.8	2,0**	1.4–3.0
		Men	3.2*	1.7–6.1	2.1*	1.1–4.3
		Women	3.2*	2.0–5.0	1.9*	1.2–3.0
	Feel that the work is physically demanding and strenuous	Total	2.8*	2.0–3.9		
		Men	2.8*	1.6–4.8		
		Women	2.8*	1.9–4.1		
	Strenuous unilateral working movements	Total	2.6*	1.8–3.8		
		Men	3.7*	1.9–7.1		
		Women	2.3*	1.5–3.6		
	Turns in the same way many times an hour	Total	2.4*	1.8–3.2		
		Men	2.8*	1.7–4.6		
		Women	2.2*	1.5–3.1		
	Work with hands raised in level with the shoulders or higher	Total	2.3*	1.6–3.3		
		Men	2.1*	1.1–4.0		
		Women	2.5*	1.6–4.0		
	Lifting at least 15 kg several times a day	Total	2.2*	1.6–2.9		
		Men	2.0*	1.2–3.3		
		Women	2.8*	2.0–4.0		
Mental work environment	Frequent feeling of own work effort being insufficient at the end of the day	Total	4.3*	3.2–5.8	3.8**	2.6–5.5
		Men	4.2*	2.5–7.1	3.7**	1.9–7.1
		Women	4.1*	2.9–5.8	3.6**	2.2–5.8
	Experience the work as restricted and with lack of freedom	Total	3.5*	2.4–4.9	2.6**	1.8–3.8
		Men	4.3*	2.4–7.9	3.5**	1.8–6.6
		Women	2.8*	1.8–4.4	2.1**	1.3–3.6
	Working alone and at risk of unsafe or threatening situations	Total	2.4*	1.8–3.2	1.8**	1.2–2.7
		Men	1.8	1.1–3.1	1.5	0.8–2.9
		Women	2.8*	2.0–4.1	2.1**	1.3–3.5
	Feel that there is far too much to do at work	Total	1.5	0.7–3.2		
		Men	0.8	0.3–2.5		
		Women	2.1	0.7–5.9		
	No ability to decide the arrangement of own work	Total	1.3	1.0–1.8		
		Men	1.9*	1.1–3.2		
		Women	1.0	0.8–1.5		
Work pace and working time	Dissatisfied with working time	Total	3.5*	2.4–5.2	2.9**	2.0–4.4
		Men	2.5*	1.2–4.9	1.9	0.9–4.0
		Women	4.2*	2.7–6.8	3.7**	2.3–6.1
	Not enough rest and relaxation between working days (apart from sleeping time)	Total	2.7*	2.0–3.6	2.3**	1.6–3.3
		Men	3.0*	1.8–5.1	3.6**	1.9–6.9
		Women	2.3*	1.6–3.3	1.7*	1.0–2.6
	Lack of opportunity to determine work rate	Total	1.9*	1.5–2.6		
		Men	1.9*	1.2–3.2		
		Women	1.7*	1.2–2.4		
Management and social support in work	Lack of opportunity to partly decide the times at which different tasks are to be done	Total	2.2*	1.6–2.9	2.3**	1.2–3.6
		Men	2.0*	1.3–3.3	1.5	0.7–3.2
		Women	2.0	1.4–2.9	2.7**	1.5–4.9
	Lack of enough support and help from superiors and colleagues at work	Total	2.1*	1.2–3.5	2.0*	1.2–3.4
		Men	1.0	0.4–2.1	1.0	0.5–2.0
		Women	3.5*	1.7–7.4	3.5**	1.7–7.3
	Lack of enough opportunities for influence at work	Total	2.1*	1.3–3.6		
		Men	1.1	0.5–2.3		
		Women	3.0*	1.5–6.2		
Motivation and work satisfaction	I am in generally dissatisfied with the work tasks	Total	5.3**	3.4–8.1	5.5**	3.4–9.5
		Men	6.6*	3.3–13.0	6.9**	2.9–16.3
		Women	4.7*	2.7–8.3	4.9**	2.4–9.9
	The work tasks are monotonous	Total	2.8*	1.9–3.9	1.6*	1.1–2.6
		Men	4.3*	2.4–7.6	2.0	0.9–4.5
		Women	2.3*	1.4–3.6	1.5	0.8–2.8
	Much of the work I do is meaningless	Total	2.3*	1.5–3.7		
		Men	2.6*	1.3–5.4		
		Women	2.5*	1.3–4.6		
Competence and possibility for skills development	The work tasks are too simple for my competence	Total	1.9*	1.1–3.2	1.9*	1.1–3.2
		Men	1.2	0.5–2.7	1.2	0.5–2.7
		Women	2.7*	1.3–5.3	2.7*	1.3–5.3
	The work gives no opportunity to learn something new and to develop professionally	Total	1.8*	1.3–2.5		
		Men	1.6	0.9–2.7		
		Women	2.0*	1.3–2.9		

For the theme Mental work environment, the variables “frequent feeling of own work effort being insufficient at the end of the day”, “experience of the work as restricted and with lack of freedom,” and “working alone and at risk of unsafe or threatening situations” were statistically significantly associated with whether the respondents could work until 65 years or not (Table 1). But the statement “working alone and at risk of unsafe or threatening situations” was only statistically significantly associated with whether the respondents could work until 65 years or not among women when the sexes were subsequently split in the multivariate model of the theme.

In the theme Work pace and working time, the statements “dissatisfied with working hours” and “not enough rest and relaxation between working days (apart from sleeping time)” were mostly associated with whether the respondents could work until 65 years or not (Table 1). The statement “dissatisfied with working hours” was only statistically significant to whether women could work until 65 years or not when the sexes were subsequently split in the multivariate model of the theme.

For the theme Management and social support in work, the statements “lack of opportunity to partly decide the times at which different tasks are to be done” and “lack of enough support and help from superiors and colleagues at work” were mostly associated with whether the respondents could work until 65 years or not (Table 1). However, those statements were only statistically significant to women when the sexes were subsequently split in the multivariate model of the theme.

In the theme Motivation and work satisfaction, the statements “I am generally dissatisfied with the work tasks” and “the work tasks are monotonous” were mostly associated with whether the respondents could work until 65 years or not (Table 1). The statement “I am generally dissatisfied with the work tasks” was mostly associated with whether both the male and female respondents could work until 65 years or not.

In the theme Competence and possibility for skills development, only two variables were included from the questionnaire. Only “the work tasks are too simple for my competence” was shown to be statistically significant to whether the respondents could work until 65 years or not when including both statements in a multivariate model of the theme (Table 1).

In the last step of the logistic analysis, all six themes were analyzed together in a joint multivariate model (Table 2). All the discarded variables were once again verified one at a time within the model to test the robustness of the final multivariate model. The final multivariate model found that the variable most highly associated with whether the individuals aged 55–64 years could work until 65 years or not was the statement “bodily exhaustion after work” in the theme Physical work environment, followed by the statements “frequent feeling of own work effort being insufficient at the end of the day,” “experience the work as restricted and with lack of freedom” and “working alone and at risk of unsafe or threatening situations” in the theme Mental work environment, and the statement “I am in generally dissatisfied with the work tasks” in the theme Motivation and work satisfaction (Table 2). After splitting the sexes in this final multivariate model, the analysis found that most highly associated with whether women could work until age 65 or not was “bodily exhaustion after work.” And most highly associated with whether men could work until age 65 or not was “frequent feeling of own work effort being insufficient at the end of the day”.

**Table 2:** Final multivariate model of all six themes and 23 items regarding the possibility of working until age 65 in the current occupation and work environmental factors. Outcome for items included in the model and the corresponding odds ratios (ORs) and 95% confidence intervals (CIs) obtained from logistic regression. Increased ORs for an item indicate an individual’s decreased possibility of working until age 65 in their current occupation. (* = *p* < 0.05; ** = *p* < 0.005)

Theme	Statement	Groups	The final total multivariate model	
			OR	95% Cl
Physical work environment	Bodily exhaustion after work	Total	37**.	6.2–2.3
		Men	27**.	5.7–1.3
		Women	47**.	9.5–2.4
Mental work environment	Frequent feeling of own work effort beingis insufficient at the end of the day	Total	29**.	4.6–1.8
		Men	35**.	8.0–1.6
		Women	24**.	4.4–1.3
	Experience the work as restricted and with lack of freedom	Total	18*.	2.9–1.1
		Men	24*.	5.4–1.0
		Women	2.1	5.0–0.9
	Working alone and at risk of unsafe or threatening situations	Total	17*.	2.7–1.1
		Men	1.2	2.6–0.6
		Women	22*.	4.0–1.2
Motivation and work satisfaction	I am in generally dissatisfied with the work tasks	Total	19*.	3.8–1.0
		Men	1.9	5.6–0.6
		Women	2.1	5.0–0.9

### Possibility of working until age 65 in the current occupation related to systematic work environment management and to occupational health care services

The European states have introduced some initiatives and regulations to decrease occupational health problems and injuries and to enable a better work environment in the societies. For example, it is compulsory under EU work environment regulations to perform systematic work environment inspections in Europe (89/39/EEG). However, compliance with this injunction is not always satisfactory at all workplaces. Access to occupational health care is also a measure that aims to improve the work environment of companies and organizations. In the next step of the analysis in this study, the relationship between the possibility of individuals aged 55–64 to work until age 65 in their current occupation and whether or not good and active systematic work environmental management was executed in their workplace as well as if they have access to occupational health care services. Active systematic work environmental management in the workplace was statistically significantly associated with whether individuals could work in their current occupation until 65 years of age (Total: OR 1.7, 95% CI 1.3–2.3; Men: OR 2.2, 95% CI 1.4–3.6; Women: OR 1.5, 95% CI 1.1–2.2). However, whether the respondents had access to occupational health care services was not statistically significantly associated with whether individuals could work in their current occupation until 65 years of age (Total: OR 1.3, 95% CI 0.9–1.7; Men: OR 1.7, 95% CI 0.9–2.9; Women: OR 1.0, 95% CI 0.7–1.5).

### Work-related difficulties and bodily disorders among men and women in Sweden

The frequency analysis of the Survey of National Work-Related Health Disorders found that spinal disorders (except neck) were the most common work-related bodily disorders among men and women in Sweden (Table 3). However, a subsequent frequency analysis of men and women separately found that sleeping disorders and arm and shoulder disorders were more common among women. Mental disorders and neck or throat disorders were also more common among women.

**Table 3:** Percent of work-related bodily disorders among men and women in Sweden

Work-caused bodily health disorders	Portion of men (%) with the health disorder	Portion of women (%) with the health disorder	Total portion (%) with the health disorder
Dorsal disorders (except neck)	37.2	31.1	33.6
Sleeping disorders	23.4	34.0	29.7
Arm, shoulder disorders	26.8	31.2	29.4
Mental disorders (not localizable)	20.4	27.8	24.8
Neck or throat disorders	11.7	20.9	17.1
Hip, leg, knee disorders	10.7	9.3	9.9
Fingers, hand, wrist disorders	9.1	9.2	9.2
Hearing disorders	8.9	8.1	8.4
Head, face disorders	6.0	9.0	7.8
Stomach, intestines, other abdominal organs	2.8	4.9	4.1
Toes, foot, ankle disorders	4.1	4.0	4.0
Disorder throughout the whole body	2.3	4.5	3.6
Cardiac, vascular disorders	1.7	2.6	2.2
Chest, breast disorders	1.5	1.2	1.3
Lungs, respiratory disorders	1.4	1.0	1.2
Abdominal or pelvic disorders	1.2	0.9	1.0
Eye disorders	0.7	0.9	0.8
Blood or hematopoietic organ disorders	< 0.1	0.9	0.5
Skin disorders	0.4	0.3	0.4

The most frequent causes of the reported work-related difficulties were stress and mental pressure in the work situation, demanding work postures, heavy manual handling as well as repeated and repetitive tasks (Table 4). The frequency analysis also showed that 90% of the respondents still worked in the same profession as when their difficulties began, despite the reported work-related bodily disorders, sickness and health problems.

**Table 4:** Reported work-related difficulties among men and women in Sweden, by percent

Causes of the difficulty	Men	Woman	Total
Stress, mental pressure	32.9	47.7	41.6
Demanding work postures	31.3	28.7	29.8
Heavy manual handling	21.8	18.7	20.0
Repeated and repetitive tasks	9.9	12.2	11.3
Noise	6.1	4.9	5.4
Difficult temperature conditions	2.3	2.0	2.1
Chemical / technical products	2.8	1.6	2.1
Threats, violence	1.6	1.6	1.6
Bullying, harassments	1.1	1.8	1.5
Inebriation	1.8	0.4	0.9
Plants and animals	0.5	0.9	0.8
Agents, contagion, infection	0.1	0.3	0.2
Other/not listed cause of work-related difficulty	40.6	43.8	42.5

## Discussion

The results presented in this study show that men and women in some ways suffer from different work-related disorders, and the most common causes of work-related difficulties were stress and mental pressure followed by physically demanding work activities. However, in women-dominated workplaces and work environments, both fewer men and fewer women reported that they could not work until 65 years of age, compared to men-dominated workplaces. This study analysis of the Swedish population shows that whether or not an individual in the 55–64 age range is able to work until age 65 or beyond is mostly associated with not being bodily exhausted after work, feeling satisfied with their own performed work at the end of the day, experiencing freedom at work and being generally satisfied with their work tasks. It was also very significant to whether individuals could work until age 65 not to be working alone and not being at risk of unsafe or threatening situations, especially to women. Those who stated that given their age, it was hard to manage their work tasks were unable to work until 65 years of age. Therefore, age and ageing need to be included and reflected on in work management if more individuals are to be able to work until an older age. The analysis also stated that proper systematic work environment management and activities in the workplace increased the possibility of working until a higher age. More measures and activities are needed.

### Work related health problems and ageing

The swAge model stated the relations between different ageing concepts and the possibility to work [4–6]. Physically demanding work environments and mentally stressful work and situations speed up biological ageing, but working at a high age is not problematic if the circumstances are good [8]. However, repeated measurements show that almost every fourth person of working age has had some physical problems as a result of their work during the past year [23]. The analysis result of work-related disorders in this population-based study in Sweden stated that spinal disorders (except neck) and hip, leg and knee disorders were more common among men as a result of their work situation. At the same time, sleeping and mental disorders were more common among women as a result of their work situation. Still, shoulder disorders and neck or throat disorders were also more common among women. A physically demanding work environment with high loads and demands or poorly designed working conditions causes wear and tear, accidents and work injuries, which also increases the risk of individuals disappearing from working life early [4–6, 8, 9]. The risk of injuries occurring also increases with increasing biological age due to deterioration of various sensory systems, such as poorer vision, hearing and sensation, and balance and reaction capacity generally deteriorate with increasing age. Signs of ageing are present in the bodily functions before the age of 40. By the age of 60, virtually everyone shows signs of biological ageing in all the bodily functions and in the interaction between brain, sensory organs, circulatory systems and internal organs [23]. This is partly due to the age of the body, but also to the diseases and injuries that the individual has suffered through life and working life [24–27]. Muscle strength decreases with increasing age, which affects work ability. About half of the workforce shows a maintained average level of work ability through life and up to the age of 63–65 [11]. Generally, it becomes more difficult to perform physically heavy and static muscle work as the capacity for oxygen uptake, the heart and the blood vessels deteriorate. The ability to pump the oxygenated blood into the body’s cells in tissues and organs and to dispose of waste products from the muscles deteriorates, which increases the risk of fatigue. It is therefore important to reflect on and include other age definitions, such as biological age as well as cognitive age and social age, alongside chronological age in the society’s decisions concerning extended working life.

### Remaining in working life until age 65 and beyond

The result shows that stress and mental pressure, demanding work postures, heavy manual handling and repeated and repetitive tasks were the most common reasons for work-caused health disorders. How these demanding work-related causes can be changed remains to be determined. An important finding to reflect on in this is that those who stated that given their age it was hard to manage their present work tasks were not able to work until 65 years of age. To continuously be in a demanding environment and circumstances increases the health risks, and ageing workers run a higher risk of developing work-related disorders [11, 12, 17, 28]. Age consequently needs to be considered. A problematic and demanding work environment can cause several and multiple health problems, especially after long exposure and increasing age. The theoretical swAge model had the intention of increasing knowledge concerning the problems and to be used as a tool in this work. The swAge model describes the coherence between different age definitions as nine determinant themes of work life participation, which are sorted into four spheres of measurement [4–6]. The first sphere of determinants and measures for a sustainable working life for all ages shows the relationship between individual health in relation to physical work environment, mental work environment and the possibility of recuperation from working time and work pace. The swAge model also indicates that these factors and themes in the first sphere are directly related to individuals’ biological ageing. Therefore, measurements solving these work-related problems need to be handled with the intention of making it possible to work until an older age, and before the retirement age statutory is postponed in the state pension system. Otherwise, those who work in physically and mentally demanding work situations probably only risk being moved to other social insurance systems resulting in individual, commercial, organizational and societal suffering. To increase the possibilities for individuals to extend their working life, the best way is to manage work environmental stress and mental pressure, demanding work postures, heavy manual handling and repeated and repetitive tasks, as well as the other demanding work environmental factors [4–6, 11, 12, 17, 28].

More governments want their citizens to work in an extended working life, but there are clear links between work environment and illness [6]. Working in a physically stressful work environment that risks causing work injuries, work accidents and musculoskeletal problems is predictive of early retirement [29]. In a stressful work environment, the risk of work accidents and work-related deaths also increases. Depression, anxiety and fatigue syndrome can be the consequences of high work demands, pressured work situations and lack of fit between the individual and the environment. For example, work that includes human suffering, vulnerability, elements of impotence, threats and violence, and organizational deficiencies are at risk for the emergence of work-related stress and ill-health, such as depression, anxiety and fatigue [25, 27]. Exposure in the physical work environment by performing work with the hands above shoulder height also causes a negative impact by increasing the pressure on the tendons in the shoulder joint, which in turn causes a lack of blood circulation to the tendons around the shoulder joint. Negative physical stresses in performing long-term static work, heavy lifting, vibration and repetitive work moments affect tendons, nerves, blood flow and muscles in the shoulders, neck and wrists [28], which in turn can result in increased pressure, swelling, edema, numbness and nerve cleavage and wear on individuals’ bodies. The exit risk from working life becomes manifold if the physically demanding work is additionally carried out in a mentally stressful, anxious and noisy environment with agitating and arduous people and little control over their own work [4–6, 11].

### Gender divided working life

The logistic regression analysis of the Swedish Survey of National Work-Related Health Disorders in an extension of this study, specified that more than two-thirds of those who stated that they could not work until ordinary retirement age actually work in female-dominated workplaces. Additionally, the analyses of men’s and women’s answers separately distinguished no statistically significant difference between whether it was women or men who reported the impossibility of working until age 65 in female-dominated workplaces. Different professions have different types of stress in their work situations, and men and women traditionally work in different occupational groups and sectors. Also, earlier reviews found that women and men who share a work environment tend to develop similar morbidity [25].

The analysis results in this study from the final joint multivariate logistic regression model showed that the possibility of working until age 65 was most highly associated with a physically demanding work situation. This was also more statistically significantly associated among women than among men when the sexes were split in the analysis of the final joint multivariate model. Likewise, other research stated that women to a greater extent than men experienced the physical work demands to be more stressful with increasing age [29]. The greater suffering in physically demanding work environments is probably partly because women generally only have about 40–80% of the muscle strength of men. Muscle strength also weakens further with age, for both men and for women. Different occupations have different work environmental problems. However, it is primarily the male-dominated professions that have achieved reduced physical strain in the work environment through technical and product solutions in recent years. In addition, women and men within the same profession often work with different types of tasks and are thus burdened differently. Women work more with unilateral and repetitive tasks or with moving people, e.g. in nursing or elderly care. But even when women and men work with the same things at the same workplace, they are nevertheless physically different because tools, protective equipment and workstations are often not adapted for women. In addition, women also generally carry out a larger proportion of unpaid work in the home, which also affects their possibility to recover.

The results of the study show that the mental work environment was mainly associated with women in terms of not being able to continue working in an extended working life. An increased sensitivity to stress and increase in stress symptoms are associated with increasing age [12]. Especially for older women, the more the work changes, the more mental symptoms increase, but the cardiovascular and respiratory symptoms also increase. Women’s work-related health problems seem to reflect a more complex work situation, with both physically demanding and mentally demanding work environmental problems. Gender segregation contributes significantly to the fact that women are at a greater risk of being affected by stress injuries [2]. Women more often work in contact professions or as human service workers, occupations which generally contain mentally stressful work situations [30, 31]. For example, earlier studies found that almost one-third of all health professionals and almost half of the nurses and nurses in psychiatry described their work situation as too mentally demanding to cope with working until they were 65 years of age [30]. Those who work in contact professions run an increased risk of developing mental illness caused by their work situation [31]. They also run a higher risk of leaving their work because of the high mental burden. The work environment and the work situation are important for an extended working life because mental illness and long-term sick absence at younger ages can influence the affected individuals’ ability to cope with their continued working life. The mental work environment and the work situation are also important for older employees, as many have reported increased sensitivity to stress at increasing ages. Health problems caused by a demanding work environment accumulate over an individual’s lifetime [32]. If stress leads to a state of illness such as fatigue syndrome, anxiety or depression, it often takes a long time to recover from, despite medical and therapeutic treatment. Is it therefore important to change the mental work environment in order to make working life more sustainable to an older age. Earlier research found better self-rated health after retirement among those who worked in a deficient mental work environment with high demands, low satisfaction and insufficient ability to perform good quality work in their duties [9]. At the same time, older employees who report that they have a good working life often show better mental and physical health than those who were retired at the same age. People who have worked in a good mental work environment and experienced appropriate control over their own lives also report that they feel younger [13]. A mentally sustainable working life until an older age is characterized by dialogue, communication, participation, a sense of coherence, clarity, confidence and control over work. Based on the results of this study analysis and those of earlier studies, it is important to perform measures to improve the mental work environment at workplaces as a step toward making it more possible to work for an extended period of time.

### Motivation to work and work satisfaction

The final joint multivariate logistic regression model in this study also included the theme Motivation and work satisfaction and the importance of being generally satisfied with the work tasks if the employees were to be able to work until age 65 or beyond. According to the swAge model, motivation and work satisfaction together with the competence and possibility for skill development is associated with cognitive ageing effects on an extended working life. Being satisfied with one’s work and profession is important for well-being and long-term health [12]. Meaningful activities and engagement lead to a sense of personal value and increase the possibility of successful ageing. In previous research, the feeling of being satisfied with one’s daily work and being noticed at work have been mentioned as important factors for an extending working life [17]. It is also better for people’s health if the reason they continue working is because they are happy with their job and devoted to their organization, as opposed to being forced to work due to financial reasons [4, 5]. Experiencing work as an important part of life and a good attitude among managers and organizations towards older employees are factors that affect people’s will to work until and also after the age of 65 [4, 5, 17]. The swAge model showed the importance of developing commercial, organizational and societal measures with the intention of increasing the possibility of stimulation and motivation at work [4–6], because it is better if people themselves feel that it is stimulating and motivating to continue working, rather than being forced into an extended working life by statutory regulations.

### Employability

Enterprises and organizations need individuals with sufficient employability in regard to health, social skills, motivation and knowledge. The analysis in this study found that, despite the reported work-related bodily disorders, sickness and health problems, 90% still work in the same profession as when they got their difficulties. Sick absence and early retirement due to ill health is just the tip of the iceberg of work-related problems. The work situation needs to be suitable for the individual’s conditions because a lot of people need to work with health problems. One problem in the labor market is that people sometimes experience the feeling of being locked-in without the control to change their situation. Lock-in can consist of different scenarios [33]. Staff can thrive in their workplace and in their profession, but for reasons of work environment and physical problems, they can find it increasingly difficult to cope with their duties and have difficulty getting any other work. There are also individuals who do not dare or do not believe they have enough skills to seek work away from their current workplace. Individuals with lacking employability had difficulties changing their position or getting a new job. Employers sometimes also stigmatize older workers and do not want to hire older employees [13, 18], and it is more difficult to get a new job after 50 years of age. The social age is crucial to individuals’ employability. A study of 11,902 employees in Sweden stated age discrimination to be the most common type of discrimination in their work situation [30]. The failure to break up and move to another workplace or other profession and remain in a problematic work situation instead also increases the risk of feeling a loss of control over life and of having poor self-esteem. The experience of feeling locked-in at a workplace without control over the situation decreases wellbeing and ultimately entails risks of ill health. Therefore, it is important to ensure that employees’ skills and health are good and that there are also tasks for persons who not are fully performing on the labor market. A lot of people are not able to fully performe day after day, this must be accepted at workplaces and in the society if more people are to be able to continue working until an older age.

### Measures to force an extended working life

In order to ensure the welfare of society in the face of ongoing demographic changes, it is crucial to implement sustainable strategies on the workplace level that integrate age aspects with working conditions and health aspects [4–6]. Because of this, the OECD, the EU, authorities, the labor market, etc. are calling for knowledge regarding predictors and strategies that can work protectively in employment conditions, so that more people can cope with an extended working life [1]. Different measures are already being used to make the work environment better. Two examples of work environment measures used in most European countries are legalizations and occupational health care. Systematic work environmental management is a part of the EU regulations (89/39/EEG) and compulsory in all the participating countries of the EU.

Very well-used, working and documented systematic work environmental management in the workplace were, in this study, highly associated with whether or not the respondents thought they could work until 65 years of age. In this study, this measure is very effective. A problem with systematic work environment management arises, however, if the managers and work environment coordinators are not familiar with different work-related health risks [6]. As a step in an EU initiative and the European Agency for Safety and Health at Work (EUOSHA) campaign, all the Swedish Work Environmental Authorities inspectors were trained in the swAge model. They then used the swAge model in their information and authority work at the Swedish workplaces to increase the knowhow on how to create sustainable and healthy workplaces for all ages. Employers, unions and HR continuously use the swAge model as a check list together with the systematic work environmental management and in their risk assessment and measures initiated at their workplaces. The swAge model or other types of tools could be helpful to understand the risks and how to handle them with different measures in order to make the workplace more sustainable for all ages.

In this study of the Swedish working population, access to occupational health care services were not statistically significant to whether the participants could work until 65 years of age or not. However, occupational health care services need to increase their activities due to the ageing workforce and participate in efforts to make the work situation sustainable for all ages. Men and women aged 56–64, for example, run six times the risk of being afflicted with long-term absence from work due to work-related injuries in comparison with 16–25-year-olds [6]. Poor working conditions with a high physical and/or mental workload, negative attitudes and insulting discrimination of elderly co-workers, as well as low influence, further increase the risk of additional absence, work-related injuries and early retirement [4–6, 9, 10, 18]. In recent years, the number of incidents of reported occupational disease has increased, particularly regarding mental illness and disorders of the musculoskeletal system. If the retirement age is postponed without measures and adjustments to the ageing workforce, there will be an increased risk of suffering and raised costs due to more work-related injury and disease [1, 7]. One of the greatest challenges for the labor market is to make working life healthy and sustainable, thereby helping more people be able to work until an older age.

### Limitations

The study population only consists of people who are employed which gives a limitation in the study, since the study could not state the results regarding people who are unemployed but potentially able to work. This is a register study and there is not any data regarding different professions, or blue-collar workers and white-collar workers. However, the random sample from the total Swedish working population made by Statistics Sweden stated a robustness of a diversity cross section from all professions in Sweden. Another limitation is regarding the self-reporting and that the health problems by that is subjective and not objectively reported. However, earlier studies stated that subjectively reported self-rated health is a better predictor than objective diagnosed health problems to the possibility of an extended working life and also to death by the health problems [34, 35].

## Conclusions

The demographic changes in society and simultaneous high average age among employees put a strain on organizations and enterprises. Many countries have introduced or proposed a general postponement of the retirement age to cope with the ageing population burden on the social system and to force the pace of economic advance. Yet the work situation and work environment are not a hotspot on most governments’, or enterprises and organizations, agendas. This needs to be changed. Healthy work situations and work environments need to be very central areas. This study showed that physical work environment, mental work environment, motivation and work satisfaction are significantly associated with the possibility of working beyond 65 years or not in a random selection of the Swedish labor force. The results gave validation to the theories in the swAge model.

Promoting an extended working life is a crucial issue for socioeconomic and economic welfare in the society, but is not only about delaying the statutory retirement age which is taking place in some countries right now. The employability until an older age is threatened if the work environment and work situation have not been arranged according to awareness of individuals function, variation and ageing or whether they can change profession and get other tasks or work. A sustainable working life is also threatened if individuals do not change jobs and occupations despite being injured and ill from their current occupation and work. This could strike back on the employability to pass people on and put them out of the working life. A cohesive, sustainable working life policy is needed for all ages to promote a sustainable working life until an older age in the future. How working life affects health, biological ageing and the possibility of working until an older age needs to be taken into consideration when calculating welfare, recruitments and postponing the age retirement. Also, the fact that fewer men and women in typically women dominated workplaces and professions stated that they could work until an older age highlights the difference between different professions and their possibilities of working in an extended working life. In this study a proper systematic work environmental management system in the workplace was more highly associated with whether individuals aged 55–64 years could work until 65 years of age or not. Because of this and the result that age was highly associated with difficulty in handling the work tasks, the swAge model could be used for an age perspective in the systematic work environment management. To increase organizational knowhow and make the systematic work environment management easier to execute in the managers’ daily work activities, the swAge model could be used as a checklist in activities related to systematic work environmental measures. It could provide a subtle and convenient nudge in the intention to increase employees’ health and wealth and make working life more sustainable for all ages.

Tools for managers, like the swAge-model, could therefore be a possible way to decrease the risk of work injury as well as increase the possibility for a sustainable extended working life.

## Data Availability

The data used in this study is handled by Statistics Sweden and is not publicly available without approval from Statistics Sweden. Our obtained access to the data analyzed in this study: Dnr. 8726333/222336. To get access permission to this data contact Statistics Sweden, the Swedish Labour Force Statistics: Contact Statistics Service Telephone + 46 10 479 50 00.

## Abbreviations

CI: Confidence interval
EU: European Union
EUOSHA: European Agency for Safety and Health at Work
OECD: The Organisation for Economic Co-operation and Development
OR: Odds ratio
P: Probability value
swAge: sustainable working life for all ages
SCB: Statistics Sweden
MONA: Microdata Online Access
